# Covid-related surge in global wild bird feeding: Implications for biodiversity and human-nature interaction

**DOI:** 10.1371/journal.pone.0287116

**Published:** 2023-08-02

**Authors:** Jacqueline Doremus, Liqing Li, Darryl Jones

**Affiliations:** 1 Economics Department, California Polytechnic State University, San Luis Obispo, California, United States of America; 2 Department of Agricultural Economics, Texas A&M University College Station, College Station, Texas, United States of America; 3 Centre for Planetary Health & Food Security and School of Environment & Science, Griffith University, Gold Coast, Australia; MARE – Marine and Environmental Sciences Centre, PORTUGAL

## Abstract

The global extent of supplementary bird feeding is unknown but has consequences for bird conservation and human well-being. Using a measure of search intensity for words related to bird feeding from Google, we document a surge of interest in bird feeding that occurred around the world after Covid-19 led to lockdowns where people stayed home: 115 countries saw an increase in bird feeding search interest. We test whether the existence of interest in bird feeding is associated with greater species richness of bird species, our proxy for biodiversity, and find the relationship is highly significant. Covid-19 lockdowns may have persistent influences on global bird populations and humans’ connection to nature.

## I. Introduction

The practice of feeding wild birds is one of the most popular nature-based pastimes in many parts of the world [1], and public interest in birds is keen [2]. As well as being an enormous international industry worth more than US$4 billion annually, it is also a significant way for people to experience a connection with nature [3, 4]. As urbanization accelerates worldwide, with over 50% expected to be living in large cities by 2050, the ability of residents to have at least some level of interaction with natural features becomes critically important. The health and well-being benefits to humans associated with a wide range of nature-related activities are becoming increasingly clear [5, 6]. The feeding of wild birds is especially significant in this context, as it is simple, relatively inexpensive, and can be undertaken in almost any setting, regardless of the level of development [7, 8]. Successfully feeding birds is possible in a high-rise apartment balcony as well as in a large, vegetated garden [9]. Furthermore, growing evidence shows that people engaging in bird feeding are more likely to have pro-environmental values and support conservation initiatives [7]. For these and other reasons, many organizations are exploring ways to enable and encourage opportunities for bird feeding [1, 10, 11].

The advent of Covid-19 has had unprecedented impacts on human activities and, subsequently, on wildlife as well, especially in urban areas [12, 13]. Numerous studies have already noted increased interest in nature-related behaviors among people suddenly restricted in their movements by Covid-19 lockdowns [14]. In several European countries, Roll et al. [15] found a substantial increase in online attention on common bird species but noted an equally marked decline in this interest soon after. Bird feeding, however, may be different, as it enables direct contact with real animals rather than digital images. Because of the intimate nature of the interaction between human and wild animal during wild bird feeding, it can be among the most effective forms of connecting with nature [8].

Backyard feeding helps wild birds survive during critical periods when foraging is difficult or frequent, such as during winter, migration, and when feeding nestlings [16]. Randomized control trials have shown that bird feeding improved the individual health of birds by increasing antioxidant levels, reducing stress, and contributing to faster feather growth [17]. On the other hand, garden bird feeding may reshape migration patterns [18], affect ecological communities [19–21] and alter bird ranges [22]. Understanding the global extent of bird feeding and its seasonal patterns is vital for understanding changes in bird populations. These connections become even more important given that accelerating changes in climate affect bird populations through lack of food availability [23].

Until recently, it was assumed that the practice of bird feeding was primarily undertaken in the Northern Hemisphere and directed at smaller species that did not migrate [24–26]. While this may account for the origins of the practice, recent studies have found bird feeding to be far more widespread than was previously thought [1, 27]. The extent and characteristics of bird feeding remain poorly understood, yet the implications for human well-being and conservation could be profound [5, 9].

In this study, we explore the extent to which wild bird feeding activity (as measured by Google search index, a valid proxy parameter from Google Trends data) occurs globally. We know from other work that interests in common bird species and bird feeding increased in response to Covid in the U.S. and some European countries during the Covid-19 pandemic [15, 28]. This study first tests whether this pattern—increased interest in bird feeding in response to Covid-19 lockdowns—holds for all countries, including those in the Southern Hemisphere. If so, Covid-19 lockdowns offer a way to *reveal* the global extent of bird feeding interest, something that is poorly understood. Bird feeding is best documented in North America, Europe, and Australia, though there are exceptions [25]. If bird feeding is common in other parts of the world, this could impact migration and disease patterns. This study is the first to document the practice of bird feeding at the level of individual countries.

In addition, we were interested in whether the practice of bird feeding was related to other factors, including the number of bird species in a country. Previous research has explored the factors that influence interest in bird feeding within a single country, such as socioeconomics and attitudes toward nature [29, 30]. One study found that the number of bird species within a U.S. state was associated with greater interest in bird feeding in response to Covid-19 lockdowns [28]. The reason for this relationship is unclear. However, if this pattern occurs globally, there may be important implications for avian communities [3].

To further our understanding of global wild bird feeding patterns, we investigated two interdependent questions: (1) was there evidence of increased interest in bird feeding and related topics at a global scale after Covid-19 lockdowns, relative to before; and (2) is species richness correlated with the level of interest in bird feeding? We use species richness as a proxy for the biological diversity of life within an area, e.g., biodiversity, while acknowledging that it is an imperfect measure [31]. Given the relationship between the practice of bird feeding, human mental health, and a variety of pro-environmental attributes, the implications are of great significance for human well-being and biodiversity conservation.

## II. Data

### a. Google Trends

Google is, by far, the most widely used search engine in the world, accounting for 90% of the worldwide search market share over the past ten years [32]. Google Trends data provided by Google supplies an index to show the popularity of terms searched in Google across various regions and languages within a given period. Therefore, Google Trends data can be considered a representative sample of internet search queries globally. Google Trends searches predict behavior change in the stock market [33], tourism [34], suicide [35], and Covid-19 spread [36]. For bird feeding specifically, Google searches for bird feeding correlate with bird feeding behavior: Brock et al. [28] use data from Cornell’s Project FeederWatch and find an increase in bird feeding after Covid-19 lockdowns in the U.S. that mirrors a similar increase in Google searches for bird feeding terms. We use Google search data to assess whether residents are interested in bird feeding, as opposed to measuring the intensity of feeding.

We used two types of data provided by Google Trends to explore global bird engagement. First, we used a panel (01/01/2019 to 05/31/2020) of weekly indexed search frequency data to examine the change in search interests over time because of the Covid-19 lockdowns. We observed the data in a nation for each week (e.g., the search frequency of a search term in the U.K. in week 30 of the year 2019). Our study period follows Rousseau and Deschacht [37], a study that explores public awareness of nature and the environment during the COVID-19 crisis using Google Trends data. The relative search intensity was calculated as the number of daily searches for the search topic relative to all other search queries in the given time and region. The Google Trends index represents the relative search intensity of a search topic over the requested time period in a geographical area relative to the highest point for the given region and time. It is scaled from 0 to 100, where 100 is the week with the most searches for that topic. If the search volume is very low throughout the period, Google Trends does not report data for that geographical area for the whole period.

Second, we used cross-sectional data to explore the search interest of a search topic by nation during the same time period (01/01/2019 to 05/31/2020). The data include countries with lower search volume, which is recommended by Google as a method to include smaller countries. This index was also calculated from 0 to 100, where 100 represents the location where the search topic was most popular during the selected time frame. A high index means a higher relative search intensity (proportion of all queries) but not a higher absolute number of queries in a given region. Countries with very low search intensity do not receive a Google Trends index. Google does not report the threshold proportion of all queries at which they stop reporting an index. For the cross-sectional data, we treat the actual search intensity as an unobserved, latent variable that is only observed when it crosses this threshold. We then re-code the Google Trends index to reflect the existence of search interest, with values that have a reported Google Trends index receiving a 1 and values without an index receiving a 0.

Topics on Google Trends were a group of terms that shared the same concept in any language, which is useful for accommodating different languages used worldwide. To create our sample for the global analysis, we focused on three bird engagement related topics: "bird feeder", "bird food", and "bird bath". Bird feeders, bird food, and bird baths are popular birdwatching equipment [38]. Households use such tools to attract more bird species to visit their home and gardens [39]. The set of countries and territories with sufficient search volume varied across topics. During the study period, 115 countries (60% of all countries) and territories had sufficient volume for the "bird feeder" topic, 79 for the "bird food" topic, and 56 for the "bird bath" topic. The counts are different for the panel analysis. The exact number of countries included in each analysis are reported in Table A3 in S1 File.

### b. Bird species richness

We obtained nation-level bird species data from BirdLife International to measure local bird species richness in each country and territory. As noted earlier, this is a proxy measure for the biodiversity of an ecosystem, and it is distinct from species diversity, which is often measured using the Shannon-Wiener Index [40]. BirdLife International [41] provides spatial maps that present the distribution of bird species across the world. We overlaid national boundaries with bird species polygons to calculate the number of bird species in each country and territory. These data categorized bird species into four groupings based on seasonality: Resident, Breeding Season, Non-breeding Season, and Passage (See Table A1 in S1 File and Fig 2).

### c. Lockdown timing

To examine how lockdowns affected people’s engagement with birds, we use data from the University of Oxford on the timing of lockdown initiation by country and territory [42]. More than 90 countries and territories implemented shelter-in-place policies at the beginning of the Covid-19 pandemic [43].

### d. Internet access

Internet accessibility can be correlated with a country’s Google search intensity as the search intensity can be higher in countries with a higher percentage of populations using the internet. We obtained internet access data on the percentage of populations using the internet in each country in 2019 from the World Bank. Internet access is utilized as control while exploring the relationship between the presence or absence of search interest in bird feeding and bird species richness.

## III. Methods

To assess how Covid-19 lockdowns affected bird feeding interest, we compared Google Trend search intensity before and after the lockdown for countries with sufficient search interest using the panel search intensity data from Google Trends. Our empirical approach was dependent upon the fact that lockdown timing varied across nations, as shown in Fig 2 Panel A.

To estimate how interest in bird feeding changed after Covid-19 lockdowns, we used an event study empirical framework common in economics. This framework estimated how bird feeding search intensity differs across time, relative to the week before a country starts its Covid-19 lockdown

$$Y_{ct}=\alpha +\sum_{k=-52}^{k=-2}\sigma_{k}Z_{ct}^{k}+\;\sum_{k=0}^{K}\tau_{k}Z_{ct}^{k}+\Gamma_{ct}+\;\epsilon_{ct}$$ (1)

The expression is indexed at the country *c* and week *t* level. $Z_{ct}^{k}$ are a set of variables that indicate the number of k weeks relative to the week a country c’s first lockdown (k = 0 is the week that the first lockdown was initiated). The σ_k_ and τ_k_ measure changes in search intensity in each week before and after the first lockdown in a county, respectively. For each week, we plotted the coefficients *σ* and *τ* for each week on a graph to indicate the average change in bird feeding before and after the week before the first lockdown, after controlling for the average bird feeding response of that week and for that country, Γ_*ct*_, and the average overall, *α*. The error term, *ϵ*_*ct*_, is unlikely to be independent across weeks for a country and instead will be correlated. For this reason, we clustered the standard errors for each country. This specification allowed us to test whether there were trends in bird feeding that predated lockdown by assessing the vector of coefficients *σ*_*k*_. This also allowed us to determine how the response evolved after lockdown by assessing the vector of coefficients *τ*_*k*_.

### a. Bird species richness and bird feeding

We tested whether countries with greater bird species richness, our proxy for biodiversity, were more likely to have high search intensity for bird feeding during our sample period. Using data on bird species by country from BirdLife International and the cross-sectional search intensity data from Google Trends, we used two-tailed, two sample t-tests with unequal variances to compare the number of bird species in places with positive search intensity (labeled as "existence of search interest") in the Google Trends sample and those without enough search volume defined by Google (labeled as "absence of search interest") within the Google Trends sample. We also plotted the distributions of species for each bird grouping by these categories in box and whisker plots to compare the medians and assess the importance of outliers, which may be hard to detect in the t-tests.

We note that these t-tests describe a correlation between high biodiversity, as measured by our proxy of bird species richness, and bird feeding. We do not make causal claims that it is bird species richness that drives bird feeding; instead, we describe an observed pattern in the data.

Other factors, such as internet accessibility and GDP, could affect a country’s existence of search interest in bird feeding using Google. We use a linear probability model to examine the relationship between the existence of search interests and bird species richness while controlling for each country’s internet accessibility.


$$S_{c}={\beta_{0}+\beta }_{1}Species_{c}+\beta_{2}Species_{c}+\beta_{2}Internet_{c}+\beta_{3}GDP_{c}+\eta_{c}$$ (2)

Where S_c_ is a binary variable that equals 1 if a country is labeled as "existence of search interest" and 0 otherwise. Spieces_c_ are binary variables for the tercile of the number of bird species. The omitted category is the country with the lowest tercile of bird species richness. The coefficients β_1_ and β_2_ indicate if, compared to countries in the bottom tercile, countries with more bird species are more likely to have search interests in bird feeding. Results are qualitatively the same when using quantiles or terciles of bird species richness.

We also note that we are not controlling for all other factors that could affect the likelihood of bird feeding in a country, including income and latitude. If bird species richness is correlated with these factors, then the relationship we describe may reflect income or latitude instead of the importance of species richness itself.

## IV. Results and discussion

### a. A surge in increase in global bird feeding interest

**Fig 1:**
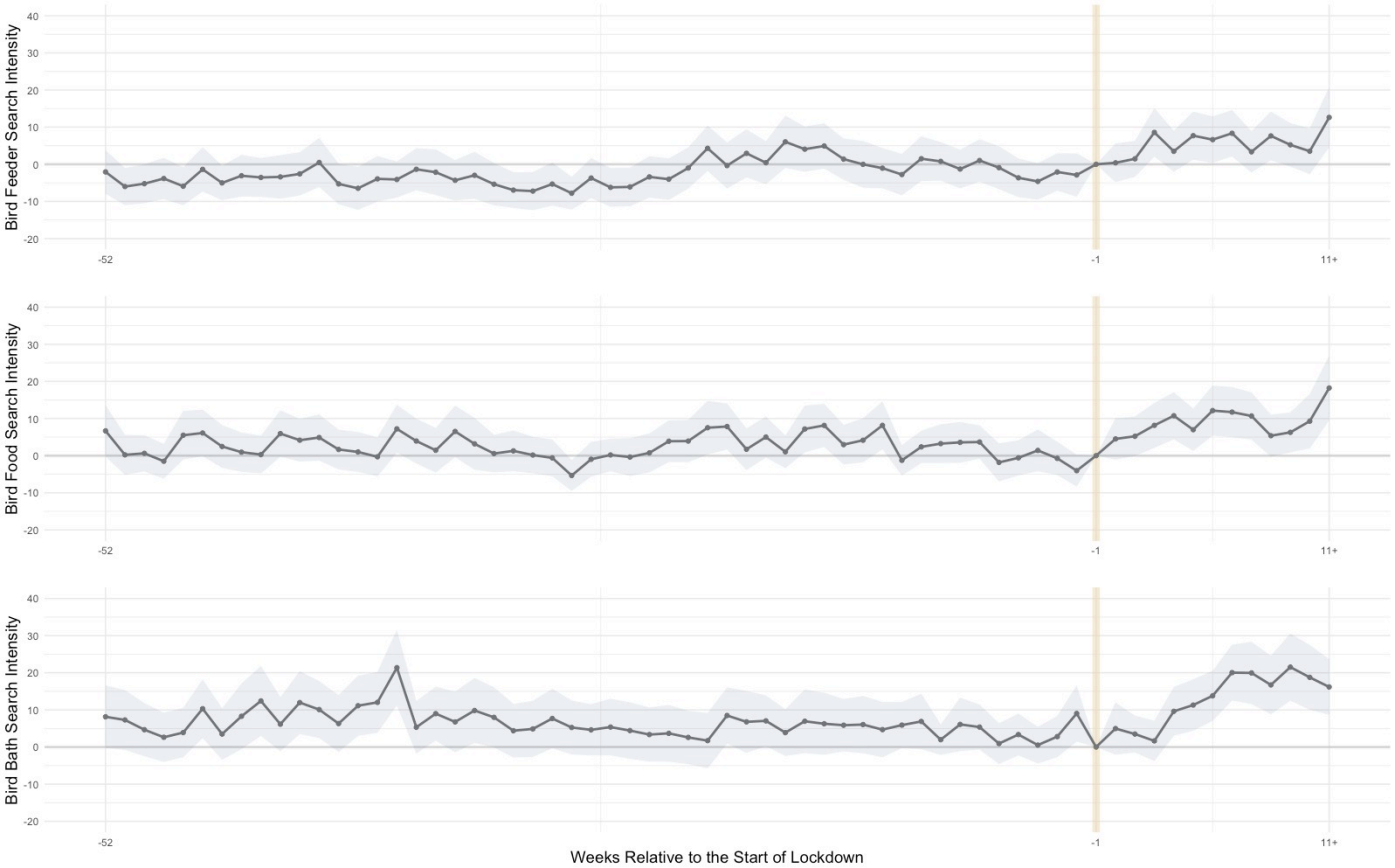
Google Trends search interest before and after a country’s first Covid-19 lockdown. Notes: This figure shows the estimated change in search intensity for the term "bird feeder," "bird food," and "bird bath" before and after Covid-19 lockdowns relative to the week before the lockdown started. We estimate this change in search intensity based on Eq (1) using data from Google Trends. The search intensity represents people’s indexed search frequency for the three terms related to bird feeding. Each point is the point estimate of the change in bird feeding interest for the period relative to the country’s first lockdown from countries with sufficient search volume for each term. The number of countries that were included for each search term: bird feeder (101), bird food (75), and bird bath (55). A list of countries included in the analysis is available in Table A3 in S1 File Column (1)-(3). A line connects each point estimate. The shaded area is the 95% confidence interval.

Our first result demonstrates that there was a significant increase in bird feeding interest occurred globally. Using countries and territories with a sufficiently high search intensity for bird feeding, bird seed, and bird baths in the panel search intensity data, we estimated the change in search intensity before and after Covid-19 lockdowns relative to the week before the lockdown started. For a period of 52 weeks prior to lockdown, search intensity was, on average, similar to what it was in the week preceding lockdown (Fig 1). The coefficients remained close to zero and within the 95% confidence interval. This is an important internal validity check as it demonstrates that there was no independent, changing interest in bird-feeding that preceded Covid-19 lockdowns.

After about two weeks of lockdown, a dramatic increase in bird feeding search intensity was evident (Fig 1). The result mirrors the interest in these topics found in the U.S. [28], a country with well-documented bird feeding interest [1]. Search intensity increased more quickly and dramatically for bird food and bird baths, doubling in intensity (see Table A2 in S1 File). This may reflect additional investments in bird feeding by people who already have a bird feeder. That said, we also observed a weak increase in search intensity for bird feeders.

The turn toward bird feeding during Covid lockdowns likely relates to changes in the relative cost of alternative forms of leisure activities, as well as increases in the benefits from connecting with nature during a stressful time. For example, Qiao et al. [44] found that participation in birdwatching was severely curtailed because of lockdown restrictions across a large number of countries. Most birdwatchers were forced to be hyper-local in their activities, restricted to their backyard or nearby park. While not a testable hypothesis in our setting, an extreme limitation on birdwatching may be a driver of bird feeding. If access to other nature-based activities was also reduced, this would make bird feeding seem relatively more attractive. Moreover, forced time at home during lockdowns may have increased opportunities for people to notice birds in their gardens and may have piqued their interest in bird feeding.

Other possible factors that may have contributed to the increase in bird feeding during the pandemic is the influence of social norms within the local communities. Research has demonstrated that households’ preferences for gardening are often associated with the norms and practices of their neighbors [45]. Thus, it is possible that the proliferation of bird feeders in local neighborhoods may have inspired others to engage in bird feeding. In addition, media coverage of the activity may also have played a significant role. For instance, the New York Times published an article about the surge in bird feeding at the beginning of the pandemic [46]. This type of media coverage may have raised awareness of and interest in the activity among a wider audience.

The benefits from bird feeding may also have grown as people sought comfort from connections with animals when lonely. People report feeling relaxed and connected to nature with more frequent bird feeding activity [8, 25]. Other papers document a pivot toward nature during the Covid-19 lockdown (e.g., Rousseau and Deschacht [37]). Thus, one possible explanation is that bird feeding interest surged because it is a popular nature-based past-time easily accessible to people at a time when nature could offer solace.

**Fig 2:**
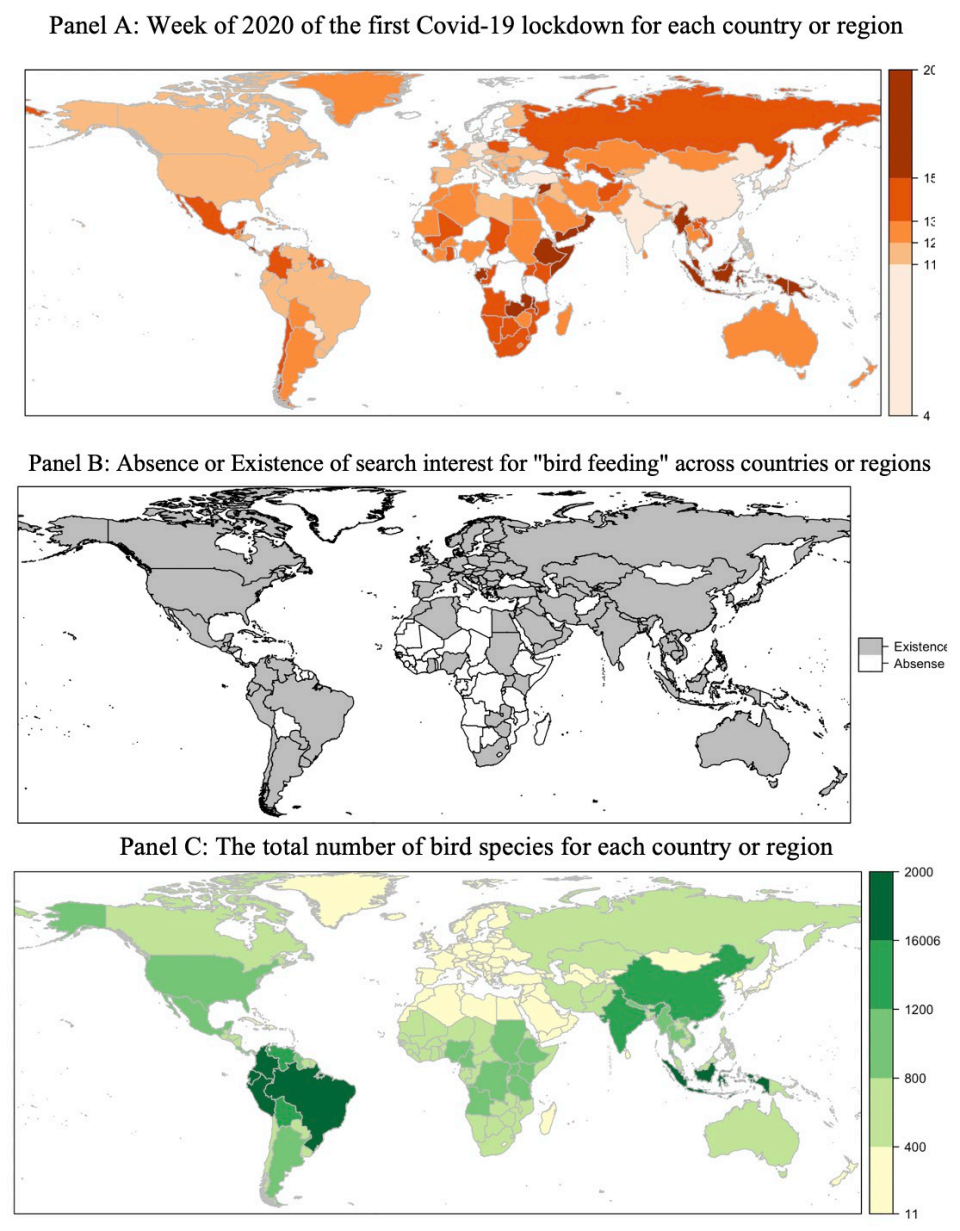
Global patterns in lockdowns, search intensity, and species richness. Notes: Panel A shows the timing of the first Covid-19 lockdown, with darker colors corresponding to later weeks in 2020 and white color represents no lockdown policies were implemented. The legend shows the number of weeks in 2020. Panel B shows the presence or absence of Google search interest for "bird feeding" across countries from 01/2019 to 05/2020. The white color represents no search. A list of these countries is available in Table A3 in S1 File Column (4). Panel C shows the total number of bird species recorded by BirdLife International for each country, with darker countries having a higher number of species. The legend represents the number of bird species in a country or region. The data source for the base map is Natural Earth. Free vector and raster map data @ naturalearthdata.com.

While increases in Google search intensity for bird feeding [28] and environmental search terms [37] in response to Covid-19 lockdowns are not new, our study used the Covid-19 lockdowns as a natural experiment to assess *global* interest in bird feeding. Fig 2 (Panel B) indicates the countries and territories that have search interest present or absent in bird feeding during our panel period, from 1/2019 to 5/2020. Given the increases in bird feeding interest, this period offers an opportunity to identify which countries and territories have a robust interest in bird feeding, though with some important limitations.

Our results show clearly that interest in bird feeding is more common around the globe than previously understood [25]. Bird feeding has historically been characterized as concentrated in Anglophone countries, including the United States, United Kingdom, and Australia, as well as in India, where it aligns with cultural practices. Indeed, the earliest recorded reference to bird feeding is likely in Hindu writings of the Vedic era, from over 3,500 years ago ([1]: 39). These countries occur in our sample, along with many other countries spanning six continents.

Variations in bird feeding across countries may be driven by cultural, geographic, or ecological reasons and is an important area of future research. In addition to cultural practices in the Indian subcontinent described above, bird feeding practices may be related to winter conditions. For example, bird feeding is more common in Northern Europe than in Southern Europe, and it remains a mostly winter-only activity in Northern Europe [1]. Indeed, there is a strong association between countries with a bird feeding policy and colder minimum temperatures [27]. However, we also found that bird feeding occurs in both the northern and southern hemispheres despite lockdowns occurring from February to April 2020, which is spring for the northern hemisphere and fall for the southern hemisphere. Bird feeding extent appears to have a wider occurrence than that described in Baverstock et al. [27], which used countries’ bird feeding policies as a proxy for bird feeding interest.

Beyond geography, bird feeding frequency may depend on socioeconomic factors. Bird feeding requires resources to share food with birds or money to buy commercial bird seed, as well as markets that supply commercial bird seed and bird feeders. Patterns of settlement within countries, including having access to parks, a balcony, or a backyard, can make bird feeding more accessible. Finally, as explored in the next section, it is possible that there are ecological features or settings that make bird feeding more enjoyable. This could include greater returns to watching birds feed and interact when doing so within a more biodiverse habitat or viewing a greater variety of species that visit a feeder.

Though the scope of feeding found by our study is wider than that of previous studies, our analysis has an important limitation. Our list of countries is likely biased toward those with higher incomes and better internet access, e.g., countries with lower income or less internet access may have *de facto* lower search intensity for bird feeding, despite local bird feeding practices. We may not be able to document this bird interest due to our measure of bird feeding, Google search intensity. The extent of bird feeding presented here may therefore be an underestimate. Despite this limitation, however, the distribution of bird feeding interest discovered in the present study demonstrates clearly that interest in bird feeding is not limited to traditional locations such as the United States, United Kingdom, Canada, Germany, and Australia [1]. Even given the limitations of our measure, among countries with high search interest, we identify some with low income and low internet access, such as Kenya and Pakistan. We encourage future work to consider how global bird feeding affects bird populations.

### b. Species richness predicts bird feeding interest

Next, we explore whether a country’s local interest in bird feeding is associated with its avian diversity. Fig 2 Panel B shows the existence and absence of search interest for bird feeding across countries or regions using the cross-sectional search intensity data, and Panel C shows how bird species richness, the total number of bird species recorded in that country, varies across the globe. The t-test results in Table 1 indicate that countries with an absence of search interest intensity had a mean of 294 (SD = 288.6) species, while countries with existence of search interest had 511 (SD = 400.5) species. This difference is statistically significant. Moreover, this pattern holds for each bird classification, including breeding, non-breeding, passage, and resident.

**Table 1:** T-test comparing bird species richness by presence or absence of search interest for "bird feeder".

		PRESENCE SEARCH INTEREST	ABSENCE SEARCH INTEREST	T STATISTICS	P-VALUE
**TOTAL**	Mean	511.2	294.9	-4.97	***
	SD	400.5	288.6		
	N	107	144		
**RESIDENT**	Mean	352.7	215.8	-3.33	**
	SD	392.9	256.6		
	N	107	144		
**BREEDING**	Mean	99.99	25.05	-7.88	***
	SD	103.2	39.1		
	N	107	140		
**NON-BREEDING**	Mean	123.9	62.67	-7.35	***
	SD	78.3	53.6		
	N	107	144		
**PASSAGE**	Mean	55	21.99	-7.46	***
	SD	43.6	23.2		
	N	106	132		

Notes:

** P < 0.01,

*** P < 0.001

Columns report the average number of species for the presence or absence of search intensity data for "bird feeder" from Google Trends. The P-value corresponds to a two-tailed t-test with unequal variance.

**Fig 3:**
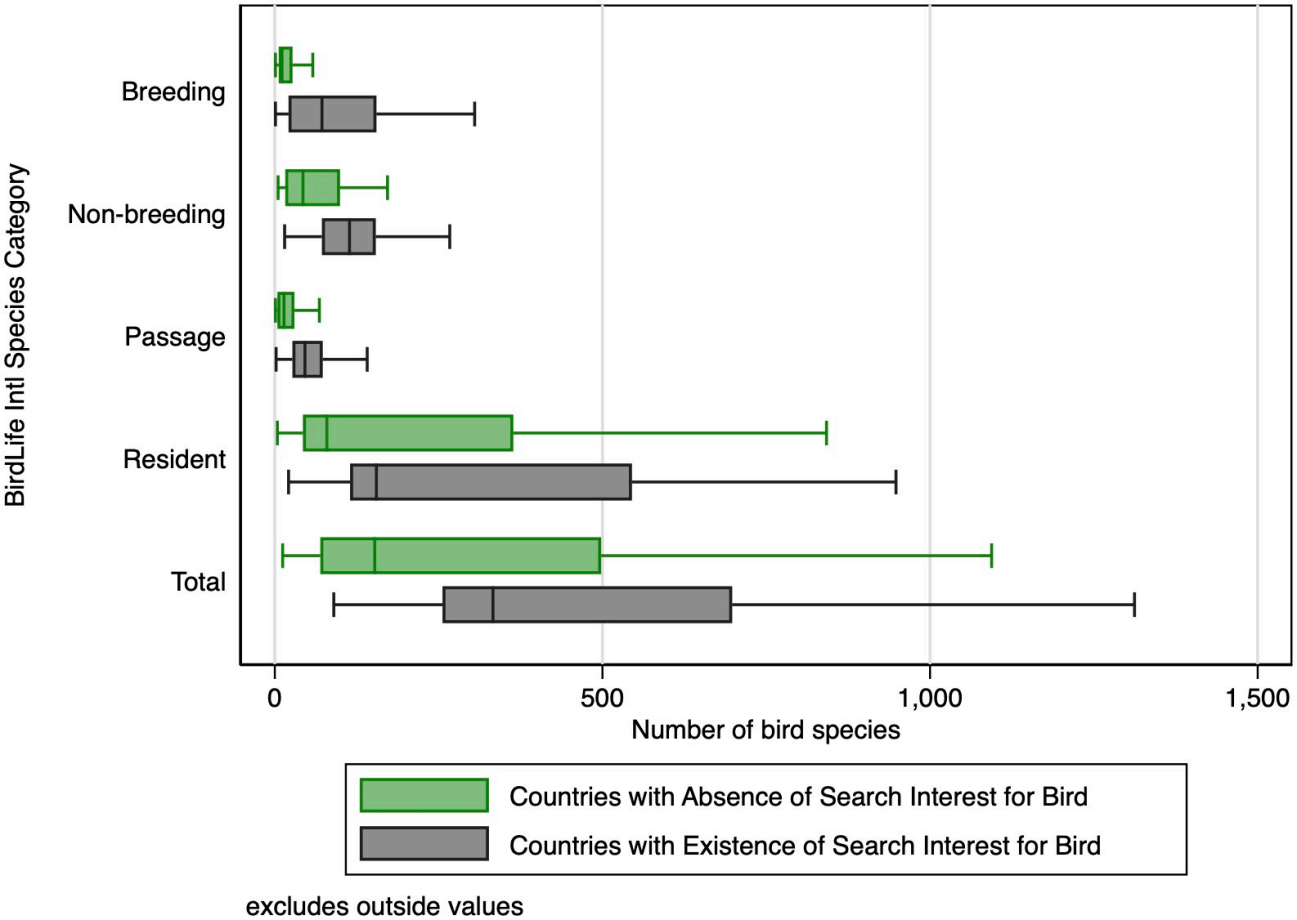
Comparing species richness across Google search interest. Notes: These are broken out by categories used by BirdLife International [41] to describe a bird’s relationship to a country, for example, whether they are a permanent resident or only visit when breeding. The line in the center of the boxplot is the median, and the ends of the box are the lower and upper quartiles. Outliers have been excluded when plotting the boxplot whiskers. The difference in bird species richness between countries with and without search interests is statistically significant for each group (see Table 1).

As a visual analog to the t-test, we compare the bird species richness of countries with an absence of interest in bird feeding to those with a presence of interest in bird feeding in Fig 3. This figure shows the distribution of species richness across countries where search interest is present or absent for "bird feeder" for each bird classification. While there is an overlap in the lower range of species richness, countries with search interest for "bird feeder" consistently have a higher median, upper quartile, and a maximum number of species for each bird classification category. It seems the distribution of species richness is shifted toward greater diversity in countries and territories where search interest is present for "bird feeder" relative to those where search interest is absent.

Results from a linear probability model indicate a positive correlation between the existence of search interest for "bird feeder" and bird species richness (Table 2 Column (1)), which confirms the finding from the t-test discussed above. Compared to countries in the bottom tercile, countries in the middle tercile are more likely to have search interests in bird feeding. Such a positive correlation still holds after we control for each country’s internet accessibility. The results in Table 2 Column (2) suggest that conditional on internet access, the existence of search interest is still positively associated with bird species richness with a slightly larger magnitude. In addition, we further analyze the relationship between these two by controlling for both internet access and GDP. The finding from this model again confirms the positive correlation between the existence of search interests and bird species richness (Table 2 Column (3)). It is important to note that the magnitude of the effects for the middle tercile gets smaller after controlling for GDP.

**Table 2:** Relationship between the existence of search interest for "bird feeder" and bird species richness.

	(1)	(2)	(3)
**Bird Species Richness (medium)**	0.480***	0.479***	0.127*
	(0.09)	(0.077)	(0.073)
**Bird Species Richness (high)**	0.121	0.443***	0.021
	(0.084)	(0.083)	(0.083)
**Internet Access**		0.010***	0.002*
		(0.0010)	(0.001)
**GDP**			0.140***
			(0.015)
**Constant**	0.364***	-0.323***	-3.010***
	(0.062)	(0.103)	(0.296)
**Observations**	172	172	172
**R2**	0.145	0.37	0.587

Note:

*p<0.1;

**p<0.05;

***p<0.01

Results show the relationship between the existence of search interest (binary, existence of search interests = 1, absence of search interests = 0) and bird species richness (binary variables for the tercile of the number of bird species) using a linear probability model.

Internet access is controlled in Columns (2) and (3), which is measured as the percentage of populations using internet in each country or territory. GDP is controlled in Column (3).

This pattern, where areas with greater species richness and, by proxy, biodiversity have more bird feeding, is new and noteworthy for two reasons. First, areas with greater species richness may also have greater ecological importance within the bird community. This could amplify both the positive and negatives of bird feeding for this community. Second, it is possible that people are sensitive to diversity, despite not being able to quantify species richness very well in surveys [47]. This sensitivity is consistent with an experiment showing that people felt better when randomly exposed to recorded bird songs along a trail and, if exposed twice, were able to attribute the increase in their well-being to greater bird presence [48]. Cox and Gaston [7] found that bird feeders preferred a greater variety of species visiting the bird feeder over a greater abundance of the same species. There may be greater well-being benefits for people in places with more diverse habitats and greater habitat complexity, which could result in more investment in bird feeding in areas with greater biodiversity [4].

## V. Conclusion and contribution

Based on the historical development of bird feeding practices, the market size for bird seed, and the literature regarding how bird feeding influences birds, one might expect feeding to be concentrated across the United States, Canada, the UK, Germany, and Australia [1]. Our results, however, assert that bird feeding is occurring at a global scale; large increases in Google search intensity after lockdowns occurred in 115 countries that had sufficient search volumes. To the best of our knowledge, this is the first paper to measure people’s interest in bird feeding at a global scale. The extensive practice of supplementary bird feeding documented in this study has broad implications for avian communities and their migratory patterns. While providing supplementary food for wild birds can be beneficial for them in terms of survival during periods of resource scarcity [16] and improved health [17], there is also evidence to suggest that bird feeding may alter ecological communities and potentially have negative effects on biodiversity [20]. It is, therefore, imperative that we understand the global extent of bird feeding in order to gain a more comprehensive understanding of its potential impacts on both avian and human well-being at a continental and global scale. Future work should further explore bird feeding patterns in parts of the world with limited formal data collection and increase the cultural and biophysical diversity of settings where local bird feeding is studied.

Given the widespread practice of bird feeding at a global scale, we sought to examine whether there is a correlation between environmental factors, such as bird species richness, and interest in bird feeding. Our analysis revealed that countries and territories with higher biodiversity, as measured by the number of bird species present, tended to have a higher level of interest in bird feeding. We also found that interest in feeding birds was shared across latitudes. As initial lockdowns occurred in February and April 2020, corresponding to spring for the northern hemisphere and fall for the southern hemisphere, this suggests a trend towards year-round bird feeding instead of winter-only [1]. Though we found a relationship between bird species richness and bird feeding, we do not claim that a greater number of bird species is *the reason* for the difference. Future work should continue to describe patterns in factors that can be associated with bird feeding interest, including socioeconomic and geographic factors, such as income and latitude, with a goal of eventually estimating causal drivers of bird feeding.

Documenting the presence and absence of interest in bird feeding is the first step to understanding global patterns. A limitation of our study is that we do not characterize how bird feeding varies across countries that show interest in feeding through Google Trends. Future work should investigate bird feeding intensity, for example, the per capita practice of bird feeding, which may vary widely across countries with interest in bird feeding as measured through Google Trends.

An appreciation of biodiversity is an important predictor for supporting conservation and environmental issues [49], yet public understanding of biodiversity is generally poor [50]. For example, a study from Switzerland found that people overestimated the number of species in their country by a factor of 30 [47]. Our paper shows that even though people may lack formal scientific knowledge of species richness, they may be more attuned than we give them credit for because bird feeding responses were higher in areas with more bird species richness. This suggests that exposure of people to wild birds through feeding may have long-term positive consequences for both human well-being and conservation through pro-environmental activities [5, 10]. Given the global scale of this phenomenon, we recommend further investigations, especially in the southern hemisphere [25].

## Supporting information

S1 File (DOCX)
