# Supplementary material for: Covid-related surge in global wild bird feeding: Implications for biodiversity and human-nature interaction
Source: PLoS One. 2023 Aug 2;18(8):e0287116. doi: 10.1371/journal.pone.0287116 (PMC10395953; doi:10.1371/journal.pone.0287116)
Supplement: S1 File — (DOCX) [file pone.0287116.s001.docx]

1. **Supporting Information**


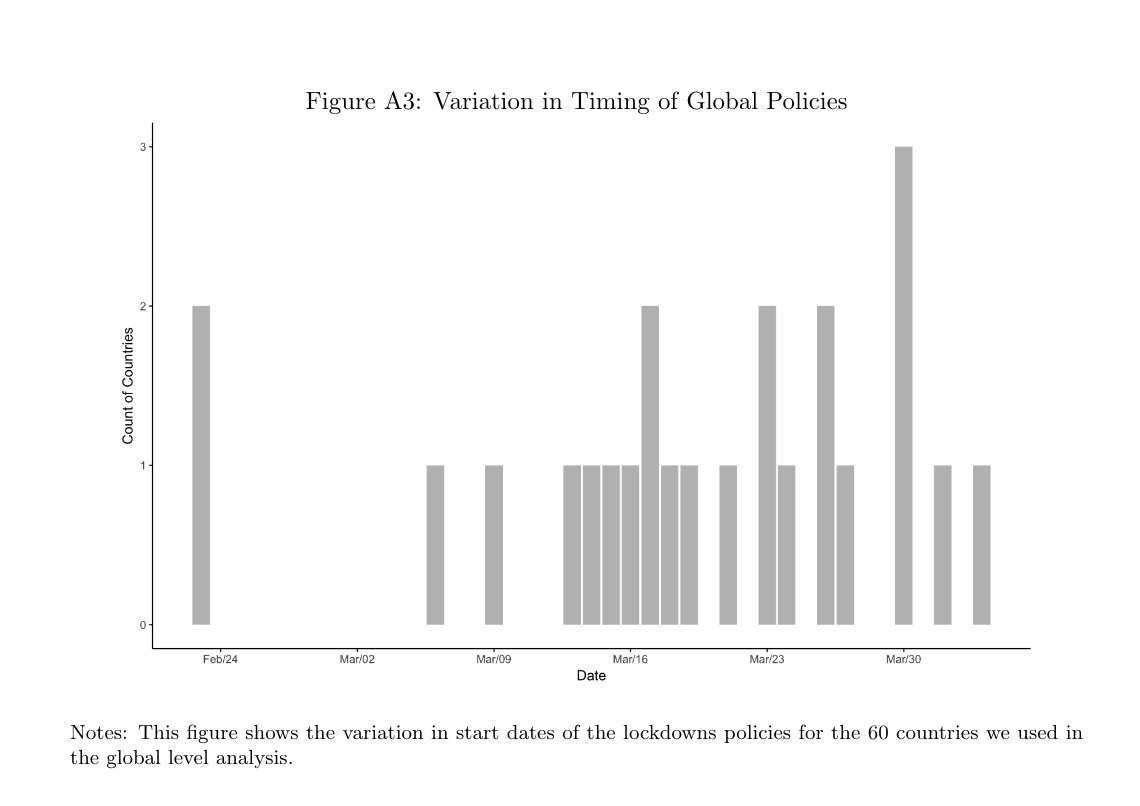


Figure 1A: Variation in timing of global lockdown policies

Table A1: Definition of different species classifications from Bird Life International

| Classification | Definition |
| --- | --- |
| Resident | The species is/was known or thought very likely to be resident throughout the year. |
| Breeding Season | The species is/was known or thought very likely to occur regularly during the breeding season and to breed or be capable of breeding. |
| Non-breeding Season | The species is/was known or thought very likely to occur regularly during the non-breeding season. In Eurasion and Norther American contexts, this encompasses ‘winter.’ |
| Passage | The species is/was known or thought very likely to occur regularly during a relatively short period(s) of the year on migration between breeding and non-breeding ranges. |

Note: Bird species classifications are obtained from Birdlife International (2020).

Table A2: Coefficient estimates for weeks after lockdown

|  | (1) | (2) | (3) |
| --- | --- | --- | --- |
|  | Bird Feeder | Bird Food | Bird Bath |
| Lockdown | 0.40 | 4.51 | 4.98 |
|  | (3.16) | (3.38) | (4.27) |
| 1 week post-lockdown | 1.47 | 5.22 | 3.50 |
|  | (2.94) | (3.23) | (3.01) |
| 2 weeks post-lockdown | 8.580** | 8.167** | 1.65 |
|  | (3.97) | (3.67) | (3.30) |
| 3 weeks post-lockdown | 3.50 | 10.750*** | 9.615** |
|  | (3.35) | (3.85) | (3.98) |
| 4 weeks post-lockdown | 7.710* | 6.986** | 11.308*** |
|  | (3.94) | (3.46) | (4.21) |
| 5 weeks post-lockdown | 6.643* | 12.139*** | 13.804*** |
|  | (3.81) | (4.08) | (4.09) |
| 6 weeks post-lockdown | 8.349** | 11.731*** | 20.039*** |
|  | (3.79) | (4.11) | (4.57) |
| 7 weeks post-lockdown | 3.36 | 10.694*** | 19.961*** |
|  | (3.38) | (3.86) | (5.08) |
| 8 weeks post-lockdown | 7.647* | 5.37 | 16.724*** |
|  | (3.99) | (3.43) | (4.79) |
| 9 weeks post-lockdown | 5.24 | 6.272* | 21.524*** |
|  | (3.56) | (3.28) | (5.49) |
| 10 weeks post-lockdown | 3.52 | 9.292** | 18.764*** |
|  | (3.74) | (4.46) | (5.30) |
| 11 weeks post-lockdown | 12.624** | 18.222*** | 16.178*** |
|  | (4.92) | (5.29) | (4.55) |
| Mean | 14.56 | 14.91 | 12.14 |
| Observations | 8510 | 5920 | 4218 |
| R2 | 0.24 | 0.26 | 0.17 |
| Note: This table shows the estimated change in search intensity for the term “bird feeder,” “bird food,” and “bird bath” after Covid-19 lockdowns relative to the country’s first lockdown*.* Standard errors clustered by country and reported in parentheses below coefficient. Coefficients correspond to τ from Expression 1. Coefficients for before lockdown, σ, also estimated but not reported. Coefficients correspond exactly to those plotted in Figure 1.  *p<0.1; **p<0.05; ***p<0.01 | | | |

Table A3: List of countries included in the analysis

|  | (1) | (2) | (3) | (4) |
| --- | --- | --- | --- | --- |
|  | Bird Feeder  (Panel data sample) | Bird Food  (Panel data sample) | Bird Bath  (Panel data sample) | Bird Feeder  (Cross-sectional sample) |
| 1 | Algeria | Algeria |  | Algeria |
| 2 | Argenti | Argenti | Argenti | Argenti |
| 3 | Australia | Australia | Australia | Australia |
| 4 | Austria | Austria | Austria | Austria |
| 5 | Azerbaijan |  |  | Azerbaijan |
| 6 | Bahrain | Bahrain |  | Bahrain |
| 7 | Bangladesh | Bangladesh | Bangladesh | Bangladesh |
| 8 | Belgium | Belgium | Belgium | Belgium |
| 9 | Bosnia & Herzegovi | Bosnia & Herzegovi |  | Bosnia & Herzegovi |
| 10 | Brazil | Brazil | Brazil | Brazil |
| 11 | Bulgaria |  |  | Bulgaria |
| 12 | Ca da | Ca da | Ca da | Ca da |
| 13 | Chile | Chile | Chile | Chile |
| 14 | Chi |  | Chi | Chi |
| 15 | Colombia | Colombia | Colombia | Colombia |
| 16 | Costa Rica |  |  | Costa Rica |
| 17 | Croatia | Croatia |  | Croatia |
| 18 | Cyprus |  |  | Cyprus |
| 19 | Czech Republic | Czech Republic | Czech Republic |  |
| 20 | Denmark | Denmark | Denmark | Denmark |
| 21 | Dominican Republic |  |  | Dominican Republic |
| 22 | Ecuador |  |  | Ecuador |
| 23 | Egypt | Egypt | Egypt | Egypt |
| 24 | Estonia |  |  | Estonia |
| 25 | Finland | Finland | Finland | Finland |
| 26 | France | France | France | France |
| 27 | Georgia |  |  | Georgia |
| 28 | Germany | Germany | Germany | Germany |
| 29 | Gha | Gha |  | Gha |
| 30 | Greece | Greece |  | Greece |
| 31 | Guatemala |  |  | Guatemala |
| 32 | Honduras |  |  | Honduras |
| 33 | Hong Kong | Hong Kong | Hong Kong | Hong Kong |
| 34 | Hungary | Hungary | Hungary | Hungary |
| 35 | India | India | India | India |
| 36 | Indonesia | Indonesia | Indonesia | Indonesia |
| 37 | Iran | Iran | Iran | Iran |
| 38 | Iraq | Iraq |  | Iraq |
| 39 | Ireland | Ireland | Ireland | Ireland |
| 40 | Israel | Israel |  | Israel |
| 41 | Italy | Italy | Italy | Italy |
| 42 | Japan | Japan | Japan | Japan |
| 43 | Jamaica |  |  | Jamaica |
| 44 | Jordan | Jordan |  | Jordan |
| 45 | Kazakhstan | Kazakhstan |  | Kazakhstan |
| 46 | Kenya | Kenya |  | Kenya |
| 47 | South Korea | South Korea | South Korea | South Korea |
| 48 | Kuwait | Kuwait |  | Kuwait |
| 49 | Kyrgyz Republic |  |  |  |
| 50 | Laos |  |  | Laos |
| 51 | Lebanon | Lebanon |  | Lebanon |
| 52 | Luxembourg | Luxembourg | Luxembourg | Luxembourg |
| 53 | Malaysia | Malaysia | Malaysia | Malaysia |
| 54 | Mexico | Mexico | Mexico | Mexico |
| 55 | Moldova |  |  | Moldova |
| 56 | Morocco | Morocco | Morocco | Morocco |
| 57 | Namibia | Namibia | Namibia |  |
| 58 | Nepal | Nepal |  | Nepal |
| 59 | Netherlands | Netherlands | Netherlands | Netherlands |
| 60 | New Zealand | New Zealand | New Zealand | New Zealand |
| 61 | Nigeria | Nigeria |  | Nigeria |
| 62 | Oman | Oman |  | Oman |
| 63 | Pakistan | Pakistan | Pakistan | Pakistan |
| 64 | Palestine |  |  | Palestine |
| 65 | Pa ma |  |  | Pa ma |
| 66 | Paraguay |  |  | Paraguay |
| 67 | Peru |  |  | Peru |
| 68 | Philippines | Philippines | Philippines | Philippines |
| 69 | Poland | Poland | Poland | Poland |
| 70 | Portugal | Portugal |  | Portugal |
| 71 | Puerto Rico |  |  | Puerto Rico |
| 72 | Qatar | Qatar |  | Qatar |
| 73 | Romania | Romania | Romania | Romania |
| 74 | Russia | Russia | Russia | Russia |
| 75 | Saudi Arabia | Saudi Arabia | Saudi Arabia | Saudi Arabia |
| 76 | Serbia | Serbia |  | Serbia |
| 77 | Singapore | Singapore | Singapore | Singapore |
| 78 | Slovakia |  |  | Slovakia |
| 79 | Slovenia | Slovenia |  | Slovenia |
| 80 | South Africa | South Africa | South Africa | South Africa |
| 81 | Spain | Spain | Spain | Spain |
| 82 | Sri Lanka | Sri Lanka | Sri Lanka | Sri Lanka |
| 83 | Sudan |  |  | Sudan |
| 84 | Switzerland | Switzerland | Switzerland | Switzerland |
| 85 | Taiwan |  |  | Taiwan |
| 86 | Thailand | Thailand | Thailand | Thailand |
| 87 | Trinidad & Tobago | Trinidad & Tobago |  | Trinidad & Tobago |
| 88 | Tunisia |  | Tunisia | Tunisia |
| 89 | Turkey | Turkey | Turkey | Turkey |
| 90 | Uganda |  |  | Uganda |
| 91 | Ukraine | Ukraine | Ukraine | Ukraine |
| 92 | United Arab Emirates | United Arab Emirates | United Arab Emirates | United Arab Emirates |
| 93 | United Kingdom | United Kingdom | United Kingdom | United Kingdom |
| 94 | United States | United States | United States | United States |
| 95 | Uruguay |  |  | Uruguay |
| 96 | Uzbekistan |  |  | Uzbekistan |
| 97 | Venezuela | Venezuela | Venezuela | Venezuela |
| 98 | Vietnam | Vietnam | Vietnam | Vietnam |
| 99 | Yemen |  |  | Yemen |
| 100 | Zambia |  |  | Zambia |
| 101 | Zimbabwe |  |  | Zimbabwe |
| 102 |  | Mauritius | Mauritius |  |
| 103 |  | Syria |  |  |
| 104 |  |  |  | Armenia |
| 105 |  |  |  | Belarus |
| 106 |  |  |  | Cambodia |
| 107 |  |  |  | Czechia |
| 108 |  |  |  | Guernsey |
| 109 |  |  |  | Isle of Man |
| 110 |  |  |  | Jersey |
| 111 |  |  |  | Kyrgyzstan |
| 112 |  |  |  | Latvia |
| 113 |  |  |  | Lithuania |
| 114 |  |  |  | Malta |
| 115 |  |  |  | Mauritius |
| 116 |  |  |  | Norway |
| 117 |  |  |  | St. Hele |
| 118 |  |  |  | Sweden |
| 119 |  |  |  | Tajikistan |
| 120 |  |  |  | Turkmenistan |
| **Count** | 101 | 75 | 55 | 115 |
| **Note:** | Column (1), (2), and (3) list countries and regions that have sufficient search volumes for the three search terms in the panel data that tracks weekly change in search interests over time from 01/01/2019 to 05/31/2020. Results based on this data are presented in Figure 1 and Table A2. Column (4) list countries and regions that have sufficient search volume for term “bird feeder” in the cross-sectional data that to explores the search interest of a search topic by nation. Result based on this data is presented in Figure 2 Panel B. | | | |
